# Exosomes confer pro-survival signals to alter the phenotype of prostate cells in their surrounding environment

**DOI:** 10.18632/oncotarget.7052

**Published:** 2016-01-28

**Authors:** Elham Hosseini-Beheshti, Wendy Choi, Louis-Bastien Weiswald, Geetanjali Kharmate, Mazyar Ghaffari, Mani Roshan-Moniri, Mohamed D. Hassona, Leslie Chan, Mei Yieng Chin, Isabella T. Tai, Paul S. Rennie, Ladan Fazli, Emma S. Tomlinson Guns

**Affiliations:** ^1^ Department of Experimental Medicine University of British Columbia, Vancouver, British Columbia, V6H 3Z6, Canada; ^2^ Department of Urologic Sciences University of British Columbia, Vancouver, British Columbia, V6H 3Z6, Canada; ^3^ The Vancouver Prostate Centre University of British Columbia, Vancouver, British Columbia, V6H 3Z6, Canada; ^4^ Division of Gastroenterology, University of British Columbia, Vancouver, British Columbia, V6H 3Z6, Canada

**Keywords:** exosomes, cancer development, cancer progression

## Abstract

Prostate cancer (PCa) is the most frequently diagnosed cancer in men. Current research on tumour-related extracellular vesicles (EVs) suggests that exosomes play a significant role in paracrine signaling pathways, thus potentially influencing cancer progression via multiple mechanisms. In fact, during the last decade numerous studies have revealed the role of EVs in the progression of various pathological conditions including cancer. Moreover, differences in the proteomic, lipidomic, and cholesterol content of exosomes derived from PCa cell lines versus benign prostate cell lines confirm that exosomes could be excellent biomarker candidates. As such, as part of an extensive proteomic analysis using LCMS we previously described a potential role of exosomes as biomarkers for PCa. Current evidence suggests that uptake of EV's into the local tumour microenvironment encouraging us to further examine the role of these vesicles in distinct mechanisms involved in the progression of PCa and castration resistant PCa. For the purpose of this study, we hypothesized that exosomes play a pivotal role in cell-cell communication in the local tumour microenvironment, conferring activation of numerous survival mechanisms during PCa progression and development of therapeutic resistance. Our *in vitro* results demonstrate that PCa derived exosomes significantly reduce apoptosis, increase cancer cell proliferation and induce cell migration in LNCaP and RWPE-1 cells. In conjunction with our *in vitro* findings, we have also demonstrated that exosomes increased tumor volume and serum PSA levels *in vivo* when xenograft bearing mice were administered DU145 cell derived exosomes intravenously. This research suggests that, regardless of androgen receptor phenotype, exosomes derived from PCa cells significantly enhance multiple mechanisms that contribute to PCa progression.

## INTRODUCTION

PCa is the most common cancer and the second leading cause of cancer-related death in men worldwide. While early detection and treatment of localized PCa has improved, many patients still die from metastatic disease. It is very well established that circulating androgens are essential for the development of both normal and malignant prostate [1] and as such the chemical removal of androgens, known as androgen deprivation therapy (ADT), remains the most effective treatment option for patients with advanced disease. However, despite an initial response to therapy, most PCas will progress to castration resistant prostate cancer (CRPC) within 2 years of treatment initiation [2–8]. CRPC progression is a complex process by which PCa cells acquire the ability to survive and proliferate in the absence of androgens. Unfortunately, currently effective chemotherapeutic agents available for CRPC improve the mean survival time of patients by only a few months [9–11]. Therefore, investigating the many diverse mechanisms involved in the progression of aggressive PCa or CRPC is essential in order to identify new therapeutic targets.

Intercellular communication is a key regulator of many physiological and pathological processes [12]. Although initially discovered by Anderson in 1969 [13], during the last several years the role of extracellular vesicles (EVs) as intercellular mediators has been an area of focus for many cancer scientists. A growing body of evidence currently demonstrates that EVs promote aggressive tumor phenotypes [14–16], angiogenesis [17], metastasis [14], drug resistance [14, 18], and can also affect the immune system [19–23].

Differences in the array of EVs found in the extracellular matrix depends largely on their cellular origin, biogenesis as well as the mechanisms associated with their formation. Exosomes are cholesterol rich EVs that are characteristically observed to be 30–200 nm in diameter [24–29]. This class of EVs, has been shown to confer changes in the surrounding cells and contribute to a refined cell communication mechanism via 1) direct stimulation of the target cells by membrane ligands, 2) receptor transfer between the donor cells and recipient cells, 3) transfer of genetic information to recipient or target cells and 4) direct stimulation of the target cells by endocytically expressed surface receptors [30, 31].

These vesicles are often found in different body fluids such as plasma [32], serum [33, 34], malignant ascites [35, 36], urine [37], amniotic fluid [38], brochoalveolar lavage fluid [39, 40] and breast milk [41]. They originate from early endosomes (EE) which later form multivesicular endosomes (MVE) that are generated upon plasmalemmal membrane budding and subsequent intracellular internalization. The resulting exosomes are rich in a plethora of various proteins [42, 43], an array of lipids, as well as nucleic acids (DNA [44, 45] and RNA [46–48]).

As a consequence of their endosomal origin, and independent of the cell type, all exosomes share some common proteins involved in membrane transport and fusion (e.g. Annexins and Flotillin), cytoskeletal proteins (e.g. Actin and Tubulin), adhesion molecules (e.g. Integrins and Tetraspanins), antigen presentation (e.g. MHC I,II), signal transduction (e.g. 14-3-3 and Syntenin) and ESCRT (Endosomal Sorting Complexes Required for Transport) components. While some of the proteins found in exosomes derived from different cell lines are the same, cellular origin of exosomes is thought to be recognizable based on their protein content [49]. For example, intestinal cell exosomes express transmembrane protein A33 on their surface, T-cell derived exosomes bear CD3 (Cluster of Differentiation 3), and prostate cell derived exosomes may be recognizable based on the presence of membrane antigen folate hydrolase 1 (FOLH1) [50].

While EV research has grown exponentially during last decade since its initial discovery by Anderson [13], cancer cell derived exosomes in particular, have been one of the main areas of interest for EV scientists, not only because of their potential source of biomarkers, but also because of their detrimental effects on immune system, which occur via blocking or inducing specific pathways and are possible as a result of their extensive range of bioactive molecules [19–23].

It is very well known that cancer cells produce many of their own growth factors in order to sustain their independent proliferative growth signalling. MAPK and PI3K/Akt pathways are recognised as the main cytoplasmic signalling components that play a central role in growth signalling [51]. Exosomes have been shown to confer changes to surrounding cells and to contribute to a refined cell-communication mechanism. In the present study, the effect of exosomes derived from androgen receptor (AR) positive or negative PCa cell derived exosomes on PCa tumour growth, progression and survival properties was investigated. We also report the effects of exosomes derived from PCa cells on the PSA level and tumor growth of mice bearing human PCa tumour xenografts after they have been systemically introduced via IV injection.

While the primary emphasis of this research was to understand the effects of different PCa derived exosomes, with distinct AR phenotypes, on cell-cell communication as they confer changes in cellular properties of neighboring cells in a tumour population, further studies are required to achieve a more precise understanding of the role of exosomes in cancer progression and metastasis at e molecular level.

## RESULTS

### Exosome purification and characterization

Exosomes were isolated and purified based on their size and density from two different PCa cell lines: LNCaP (AR +ve) and DU145 (AR –ve). Transmission electron microscopy, western blot analysis and NanoSight tracking analysis (NTA) were used to characterize their integrity and morphology, purity and size distribution.

### Transmission electron microscopy (TEM)

**Figure 1:**
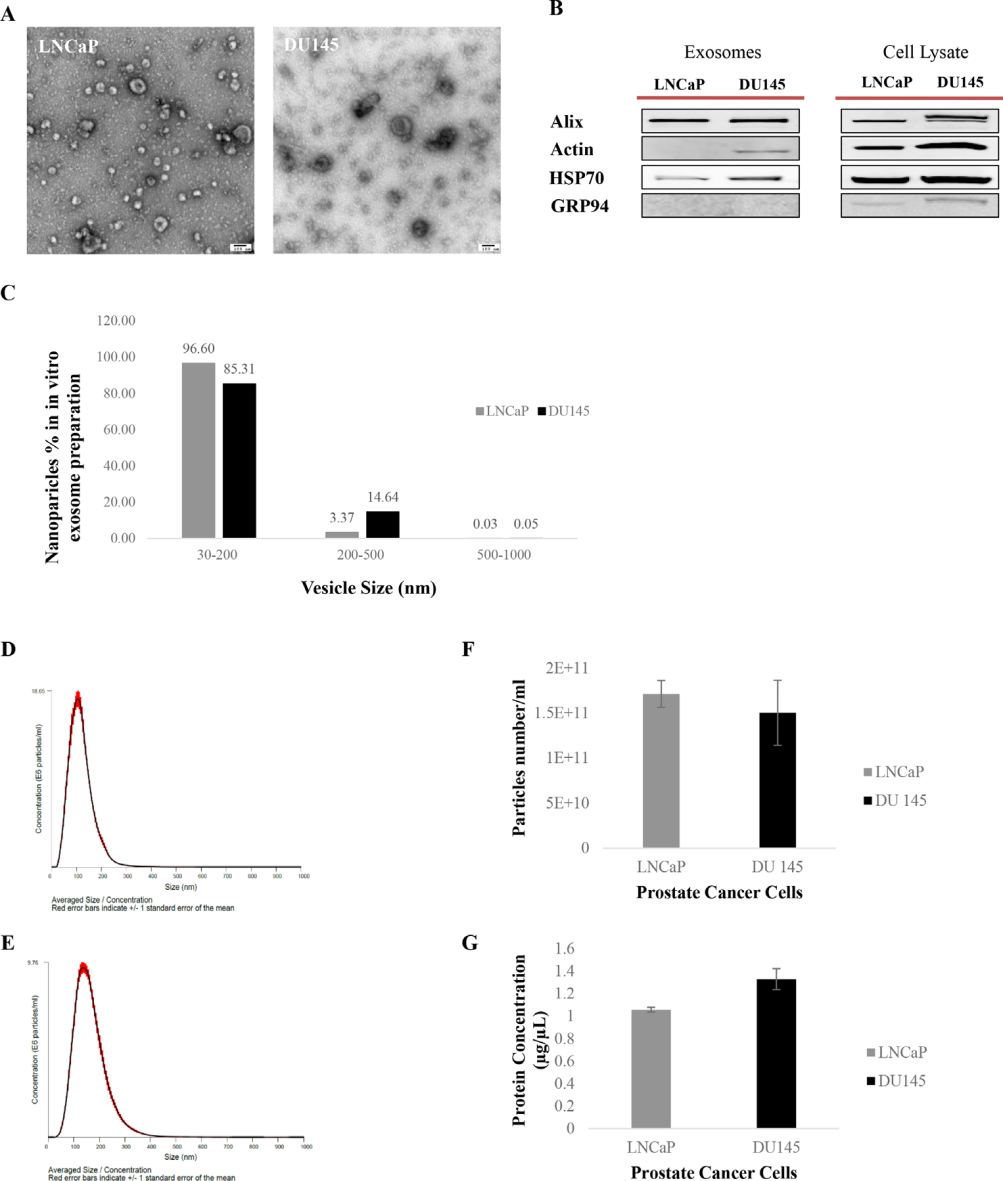
Exosome characterization (**A**) Transmission Electron Microscopy (TEM). TEM images of exosomes derived from androgen sensitive and independent prostate cancer cell lines; LNCaP and DU145. Exosomes were negatively stained with 2% uracyl acetate after removing the extra moisture. Cup-shaped structures, with 30–200 nm size were identified as being exosomes. (**B**) Western Blot analysis for exosomes marker in exosomes and cell lysate samples. Exosomes have been purified based on their unique size and density by ultracentrifugation with 30% sucrose-deuterium. Thirty micrograms of total protein associated with purified exosomes or cell lysate were analyzed by Western Blot using different exosome markers in both cell lines. (**C**) Nanoparticle Tracking Analysis. Bar chart showing the average percentage of nanoparticles within 30–200 nm, 200–500 nm, and 500–1000 nm size in *in vitro* exosome preparation. Size distribution of exosomes derived from (**D**) DU145 and (**E**) LNCaP were measured by nanoparticle tracking analysis (NTA) showed a peak at 117 +/– 0.3 nm (LNCaP) and 164 +/–1.0 nm (DU145). Bar Chart showing the (**F**) particle number/ml for both PCa Cell lines. (**G**) Protein Concentration of exosomes derived from DU145 and LNCaP Cell lines. Values are mean ± standard deviation, all values are representative of at least three independent experiments with four replicates.

To evaluate their integrity and morphology, 2.5 μl of a diluted exosome sample was loaded and fixed onto formvar-coated carbon EM grids and visualized by TEM after staining with 2% uracyl acetate as previously described [52]. TEM observation showed a very homogenous exosome mixture with a typical cup-shaped and round morphology with a diameter range of 30–200 nm (Figure 1A).

### Western blot analysis

Western blot analysis was used to identify the presence or absence of a selection of exosomal and endoplasmic reticulum (ER) markers to confirm the efficiency of our exosome isolation protocol as well as the purity of the exosome isolate. The presence of at least two or all the exosomes markers from three different categories including Alix (Anti-Apoptosis), Actin (cytoskeleton) and HSP70 (Heat-Shock Protein) alongside the absence of GRP94 (ER marker) in our Western blot data confirmed the purity of the exosomes isolated from both PCa cell lines studied (Figure 1B).

### NanoSight tracking analysis (NTA)

NTA was used to characterize the size and estimated number/ml of isolated nanoparticles from both cell lines. To better measure the purity of our exosome isolate, the percentage of larger nanoparticles with diameters between 200–500 nm and 500–1000 nm, contained within our exosome samples (nanoparticle size range: 30–200 nm) were calculated. As shown in Figure 1C the exosome isolation protocol explained in this study, which is based on size filtration and ultracentrifugation (100,000 g sedimentation force) on a 30% sucrose cushion (density), purified 85–97% nanoparticles with size of 30–200 nm, 3–15% of nanoparticles with diameters of 200–500 nm, and maximum of 0.05% of nanoparticles larger than 500 nm (500–1000 nm).

Figure 1D and 1E show the average size distribution of nanoparticles isolated using our isolation technique. In agreement with others [53, 54] peaks at 117 nm and 164 nm for nanoparticles isolated from LNCaP and DU145 respectively were observed, which are within the 30–200 nm size range characteristic of this class of EVs. The average number of nanoparticles/ml measured using the NTA system was 1.7 × 10^11^ for LNCaP and 1.5 × 10^11^ for DU145 (Figure 1F) (Data were compiled from five measurements per biological replicates (*n* = 3)).

Protein concentration of exosomes was measured using a BCA assay (Figure 1G). While the protein concentration of LNCaP cell derived exosomes appeared to be lower than DU145 cell derived exosomes, no significant differences were determined for either the number/ml of nanoparticles or protein concentration between exosome isolates from these AR +ve or –ve cell lines.

### Exosome uptake

**Figure 2:**
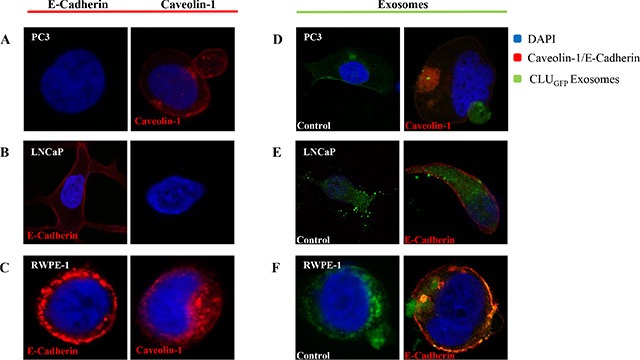
Confocal microscopy Confocal microscopy was used to visualize freshly isolated exosomes derived from a CLUGFP stably over-expressing LNCaP cell line, which contains CLUGFP, being taken up by (**A**) and (**D**) PC3 (AR-ve) and (**B**) and (**E**) LNCaP (AR +ve) PCa cell lines versus (**C**) and (**F**) benign epithelial prostate cell line RWPE-1, after overnight incubation. Both cell lines were further fixed and stained with DAPI and E-Cadherin/Caveolin-1 prior to imaging of the cells by confocal microscopy.

After cells were fixed using MeOH/Acetone to distinguish the cellular structure, all three cells were stained with DAPI (Blue, Nucleus) as well as Caveolin-1 and/or E-Cadherin (Red, Cell membrane) prior to imaging using confocal microscopy (Figure 2A, 2B, and 2C). Our results show that PC3 and RWPE-1 were stained positive for Caveolin. In fact, secretion of a huge EV rich in Caveolin was observed as captured in the PC3 cell image (Figure 2A), while in contrast LNCaP were only stained positive for E-cadherin.

To investigate the uptake and intercellular localization of exosomes, cells were incubated with 100 μl of CLU_GFP_ tagged exosomes for 12 hours (overnight) at 37°C (Figure 2D, 2E, and 2F). As can be seen in the left panel of Figure 2 and in agreement with what we observed previously [52] exosomes have been taken up by both PCa cell lines as well as the benign RWPE-1 cells regardless of their AR phenotype. Upon uptake of exosomes, the invagination of the cell membrane can be clearly seen in images of the PC3 and RWPE-1 cell lines (Figure 2D and 2F).

### *In vitro* functions of prostate cancer derived exosomes

#### Apoptosis

**Figure 3:**
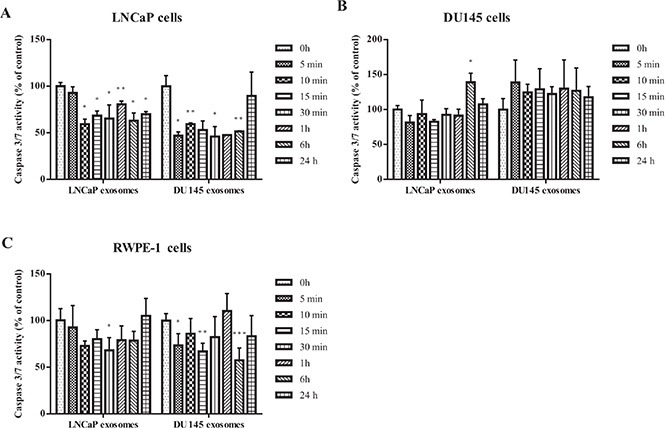
Apoptosis assay Analysis of apoptosis in PCa Cell lines (LNCaP and DU145) and benign epithelial prostate cell (RWPE-1) after treatment with 100 μg/mL of exosome derived from LNCaP or DU145 cells after 0, 5, 10, 15, 30 min, 1, 6, 24 hours. (**A**) Both DU145 and LNCaP-derived exosomes significantly reduced the caspase 3/7 activity in LNCaP cell line in most of the time points. (**B**) Exosomes derived from DU145 or LNCaP cells did not significantly influence the caspase 3/7 activity in DU145 cells. (**C**) Treatment with DU145-derived exosomes led to a significant reduction of apoptosis in RWPE-1 cells whereas the RWPE-1 cells did not display significant decrease of apoptosis after treatment with exosomes derived from LNCaP cells. All values are representative of at least two independent experiments with similar results, and are presented as the percentage of caspase 3/7 activity, where non-treated cells were regarded as 100% (*P* < 0.05).

The effect of exosomes on apoptosis in cancer cells was assessed by measuring the activities of caspase 3 and caspase 7, two of the key effectors in the apoptosis pathway. Caspase 3/7 activity was detected after LNCaP, DU145 and RWPE-1 cells were treated with exosomes derived from LNCaP or DU145 cells for 5, 10, 15, 30 minutes, 1, 6 and 24 hours. Our results show that while LNCaP and DU145 derived exosomes significantly reduce the caspase activity in LNCaP treated cells at almost all the time points and regardless of the exosomes source (Figure 3A), none of the exosome treatments (LNCaP or DU145) significantly influence apoptotic activity of DU145 cells (Figure 3B).

A similar phenomenon was seen with RWPE-1 cells treated with LNCaP-derived exosomes. Specifically, DU145-derived exosomes seem to be more effective in reducing the caspase 3/7 activity in the benign epithelial prostate cell line compared to LNCaP-derived exosomes (Figure 3C).

#### Proliferation

To determine the effect of exosomes on proliferation, the Real Time Cell Analysis system (xCELLigence, ACEA) was used to measure cellular proliferation in real time without the incorporation of labels [55].

To determine the optimal seeding concentration for all three cell lines, LNCaP, DU145 and RWPE-1 cells were seeded at numbers ranging from 2,500 to 40,000 cells/well. Cell adherence and time required for maximum density were then automatically monitored every 10 minutes for 72 hours to obtain the optimal cell seeding density.

**Figure 4:**
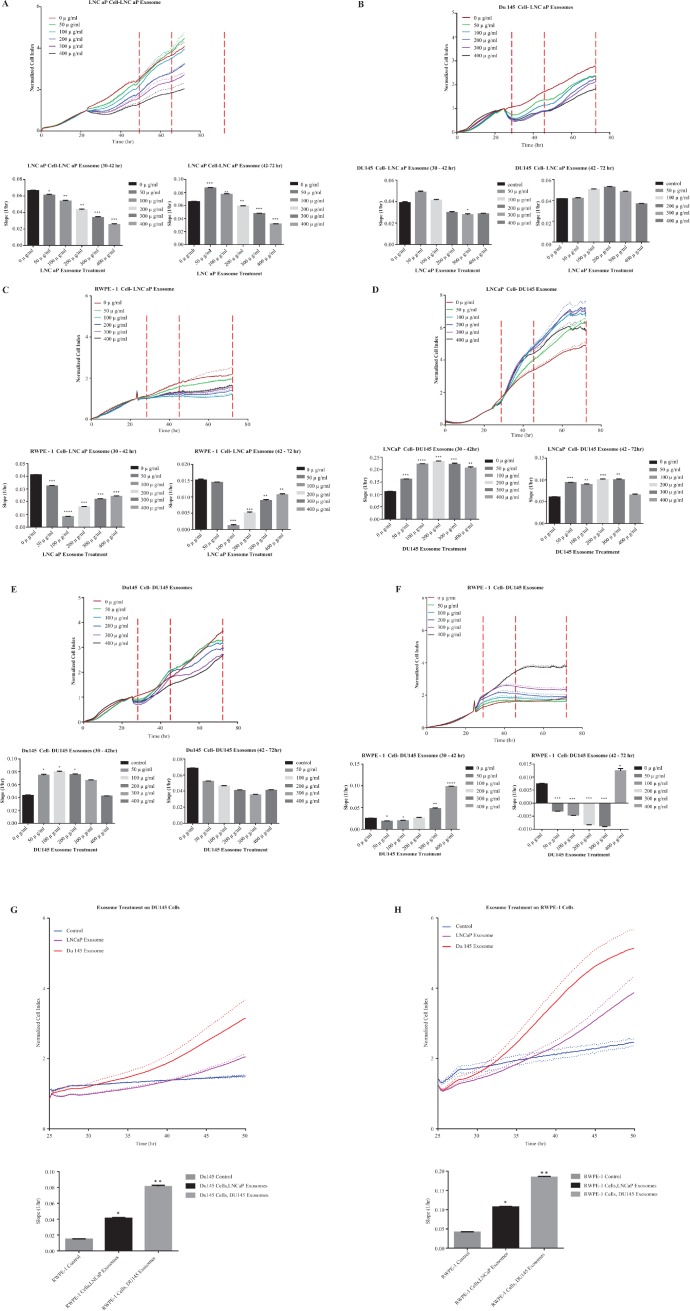
Real time proliferation and migration cell analysis Cell growth of (**A**) LNCaP, (**B**) DU145 and (**C**) RWPE-1 cells were analysed using the xCELLigence system which relies on the generation of electrical impedance as cell growth by 16-well plates were used in the impedance based system, cells were seeded at specific densities (LNCaP, RWPE-1 20,000/well, and DU145 7,000/well) after 24 hours, cells were treated with different final concentrations of the LNCaP exoxomes. All slopes were compared with the control (black bar, and red lines in the graphs at *P < 0.05, **P < 0.01, ***P < 0.001 *N* = 2. Slope was calculated by using the RTCA 2.0 software (ACEA)) Cell growth of (**D**) LNCaP, (**E**) DU145 and (**F**) RWPE-1 cells were analysed using the xCELLigence system which relies on the generation of electrical impedance as cell growth by 16-well plates were used in the impedance based system, cells were seeded at specific densities (LNCaP, RWPE-1 20,000/well, and DU145 7,000/well) after 24 hours, cells were treated with different final concentrations of the DU145 exoxomes. All slopes were compared with the control (black bar, and red lines in the graphs (*P < 0.05, **P < 0.01, ***P < 0.001, *N* = 2) (**G**) DU145 and (**H**) RWPE-1 cells were treated with 100 μg/mL of LNCaP or DU145 derived exosomes. Effect of exosome treatment on migratory properties of DU145 and RWPE-1 cells were determined using xCELLigence technology with CIM-16 plates. 20,000 cells were seeded per well and treated with exosomes after 24 hr. All values are representative of at least 4 independent experiments with similar results, and are presented as cell index on the top, and slope of the lines in the bar graphs *P* < 0.05, **P < 0.01 *n* = 4.

Three different prostate cell lines (LNCaP, DU145 and RWPE-1) were treated with 0–400 μg/ml of exosomes derived from LNCaP or DU145 cells for up to 72 hours. The assessment of cell proliferation revealed a biphasic response that was concentration dependent. LNCaP derived exosomes enhanced proliferation in both LNCaP and DU145 cells when compared to control. Specifically, the real time cell analysis demonstrated that 50 and 100 μg/ml of LNCaP exosomes could significantly increase the proliferation rate of LNCaP cells (42–72 hour), while no significant effects were seen on DU145 cells when treated with LNCaP cell derived exosomes at the range of concentrations tested. (All slopes were compared with the control, *P < 0.05, **P < 0.01, ***P < 0.001, *n* = 2) (Figure 4A and 4B).

Importantly, when RWPE-1 cells were grown in the presence of 0–400 μg/ml LNCaP exosomes, there was a significant reduction in the proliferation rate of this benign epithelial prostate cell line when treated with almost all of concentrations of LNCaP cell derived exosomes (except 50 μg/ml at 42–72 hr) (Figure 4C). We also investigated the effect of DU145 exosomes on all the three cell lines (LNCaP, DU145 and RWPE-1). As may be seen in Figure 4D almost all concentrations of the DU145 derived exosomes significantly increase LNCaP cell proliferation (except 400 μg/ml at 42–72 hour). Whereas, DU145 cell proliferation was not affected by the treatment with its own exosomes (Figure 4E).

Interestingly, DU145 exosomes has a reverse effect on RWPE-1 cell proliferation as compared to ‘no treatment’ which is similar to that which was observed following LNCaP cell derived exosome treatment (Figure 4F). Exceptionally, treatment with the 400 μg/ml DU145 exosomes, significantly increase the RWPE-1 cell proliferation.

#### Migration

Using the real time cell analysis system we also investigated whether LNCaP or DU145 cell derived exosome treatments promote the migration of DU145 and/or RWPE-1 cells (LNCaP cells were shown not to migrate in this system (data not shown)). Similar to the proliferation assay, the effect of exosomes on DU145 and RWPE-1 cells were monitored. As described above, 100 μg/ml of exosomes were added to each chamber and the real time migration of each cells from the upper chamber to the lower chamber were monitored over 48 hours. As indicated in Figure 4G both LNCaP and DU145 exosomes significantly promote the migration of DU145 cells when compared to the control (*P < 0.05, **P < 0.01, *n* = 4). Importantly, DU145 cell derived exosomes have a greater effect on the migratory properties of DU145 cells as compared to LNCaP cell derived exosomes. To test the effect of PCa derived exosomes on the benign epithelial prostate cell line, RWPE-1, 100 μg/ml of LNCaP and DU145cell derived exosomes were added to RWPE-1 cells in culture. Both of the PCa cell derived exosomes significantly increased the migration of RWPE-1 cells. Similar trends were seen in the migration slope of RWPE-1 cells when compared to DU145 cells. In both cases, the DU145 cell derived exosomes had a greater effect (almost twice that of LNCaP cell derived exosomes) on migratory properties of both cell lines (Figure 4H). Specifically, the migratory effects of both exosome treatments (LNCaP and DU145 exosomes) on RWPE-1 cell were more than 2 times higher when compared to DU145 cells.

#### Cell motility

**Figure 5:**
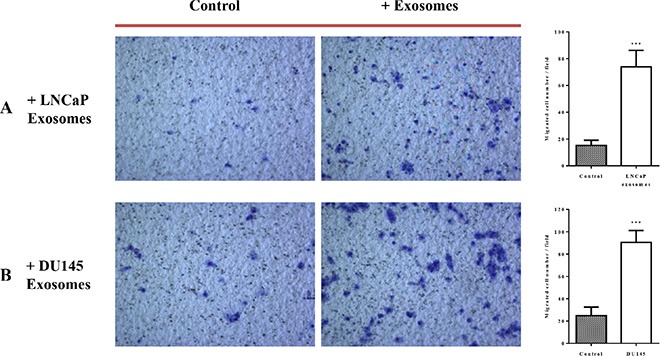
Exosomes increase RWPE-1 cell migration RWPE-1 cells were incubated for 48 hours with exosomes (100 μg/ml) derived from (**A**) LNCaP, (**B**) DU145 cells or corresponding serum-free medium and loaded into the upper chamber of a transwell. Exosomes concentrations (100 μg/ml) or serum free-medium were maintained in upper and lower chambers. After 24 h incubation, the migration activity was quantified by counting the migrated cells on the lower surface of the membrane of at least five fields per chamber using a x10 objective. Representative photographs are shown in the left panel. Quantification of migrating cells is shown in the right panel. All values are representative of at least two independent experiments with similar results, and are displayed as mean ± SD, where ***P < 0.001.

**Figure 6:**
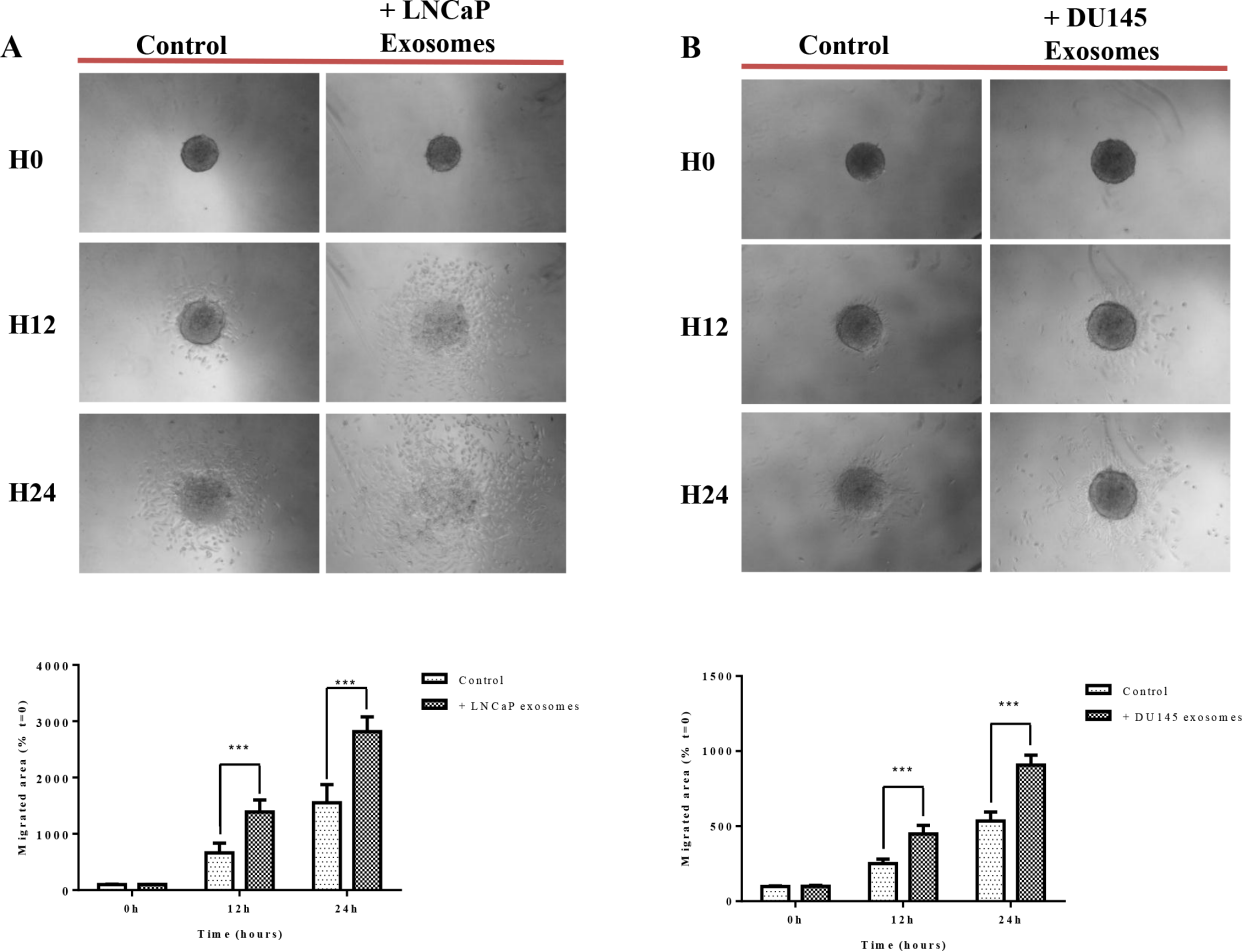
Exosomes increase RWPE-1 cell migration on collagen (I) RWPE-1 spheroids were incubated for 48 hours with exosomes (100 μg/ml) derived from (A) LNCaP, (B) DU145 cells or corresponding serum-free medium and transferred to microwells coated with type I collagen The cell migration was scored at *t* = 12 hours and *t* = 24 hours by measuring the migrated cell area and normalizing to the migration seen at *t* = 0. Representative photographs are shown in the upper panel. Quantification of migration area is shown in the lower panel. All values are representative of at least two independent experiments with similar results, and are displayed as mean ± SD, where ***P < 0.001.

The effects of exosomes derived from both PCa cell lines on the migration properties of RWPE-1 cells was also confirmed using both transwell plate (Figure 5) and tumor spheroid-based migration assays (Figure 6). RWPE-1 cells were allowed to migrate to the underside of the chamber in the presence of 100 μg/ml of LNCaP or DU145 derived exosomes or serum free medium in both chambers using fetal bovine serum as the chemo-attractant. Treatment with exosomes derived from LNCaP (Figure 5A) and DU145 (Figure 5B) cells resulted in a significant increase in the number of cells that migrated through the membrane pores.

#### 3D migration

Studying cancer cells in three-dimensional (3D) models that bears more similarity to *in vivo* tissue structures [56]. When cultured on agarose, the RWPE-1 cell line is able to form spheroids, a model considered to mimick micrometastasis or inter-capillary micro regions of solid tumors. In this study, we used the tumor spheroid-based migration assay described by Vinci *et al*. [57]. This assay attempts to mimic tumor cells spreading from a solid micro-tumor or micrometastasis. Spheroids were transferred onto type I collagen-coated microwells and migration was scored after 12 and 24 hours by measuring the migrated cell area in the presence of 100 μg/ml of LNCaP or DU145 exosomes or serum free medium. Our data showed that after 12 hours of incubation with exosomes derived from LNCaP (Figure 6A) and DU145 (Figure 6B) cells, RWPE-1 cells have a significantly greater propensity to disseminate from the spheroid, validating the results obtained using 2D standard migrations assays.

#### Pathway analysis

**Figure 7:**
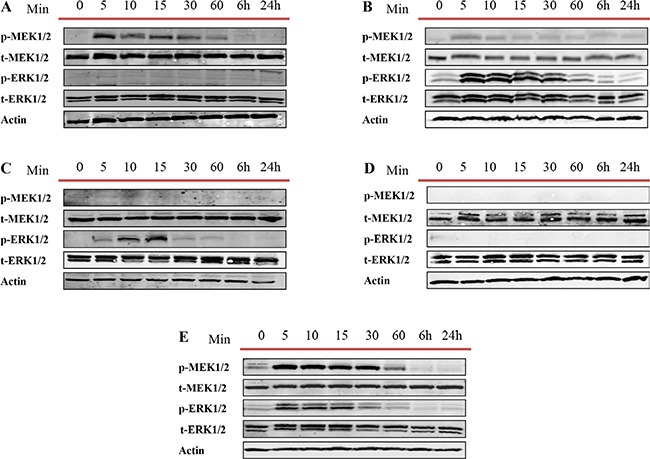
Western blot analysis (WB) Five different prostate cell lines, including (**A**) PC3, (**B**) DU145 (androgen independent), (**C**) LNCaP, (**D**) C4–2 (androgen sensitive) and (**E**) RWPE-1 (benign epithelial prostate cell line) were treated with one dose of 100 μg/mL of LNCaP derived exosomes for 5, 10, 15, 30 and 60 min, 6 and 24 hr. as indicated. Western blot was used to analyse cell lysates with the indicated antibodies.

Overexpression of the MEK/ERK pathway has been associated with CRPC and poor prognosis [58–60]. While the mechanism of activation of this signaling cascade in PCa is not fully understood we attempt to assess whether the observed decrease in apoptosis or increase of proliferation and migration after treatment of cell lines with exosomes correlates with MEK/ERK activation. We examined the activities of MEK1/2 and ERK1/2 at different time points (5, 10, 15 and 30 minutes and 1, 6 and 24 hours) after PC3, DU145, LNCaP, C4-2 and RWPE-1 cells were treated with 100 μg/ml of LNCaP cell derived exosomes. As shown in Figure 7, 100 μg/ml of LNCaP exosomes increased the expression of p-MEK1/2 and p-ERK1/2 in PC3, DU145 and RWPE-1 (Figure 7A, 7B, and 7E). While both DU145 and RWPE-1 cells demonstrated an increase in the p-ERK1/2 phosphorylation followed by p-MEK1/2 activation, the levels of p-ERK were equivalent to control for all time points studied for PC3 cells treated with LNCaP cell derived exosomes. Interestingly, while only the level of p-ERK1/2 increased in LNCaP cells after only 5 minutes treatment with LNCaP cell derived exosomes, the MEK1/2 and ERK1/2 levels did not change in C4-2 cells compared with the zero-time point (control) (Figure 7C and 7D).

**Figure 8:**
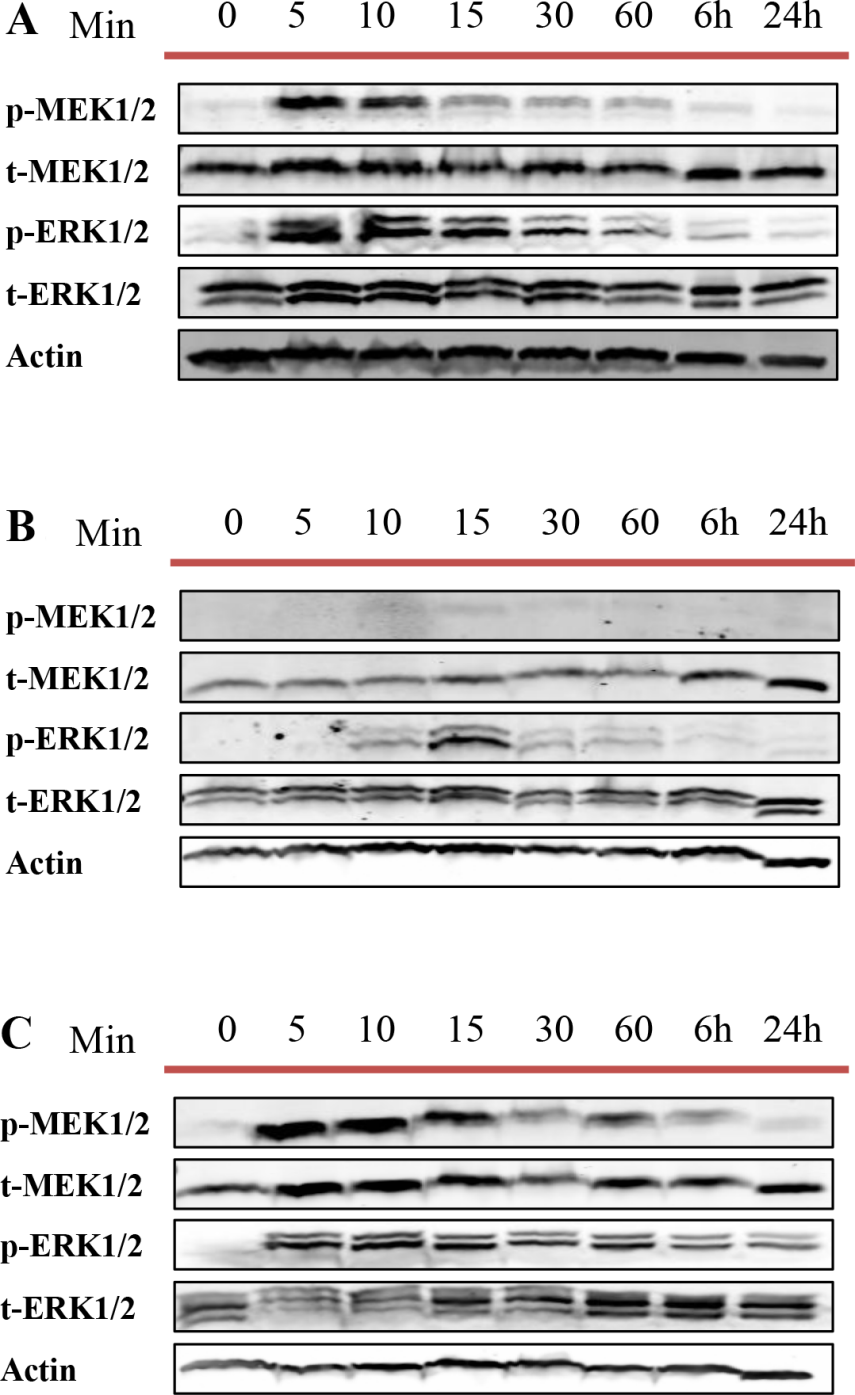
Western blot analysis (WB) Three different prostate cell lines, including (**A**) DU145 (androgen independent), (**B**) LNCaP androgen sensitive (prostate cancer cell line) and (**C**) RWPE-1 (benign epithelial prostate cell line) were treated with one dose of 100 μg/mL of DU145 derived exosomes for 5, 10, 15, 30 and 60 min, 6 and 24 hr as indicated. Western blot was used to analyse cell lysates with the indicated antibodies.

We then repeated the same experiment for DU145, LNCaP and RWPE-1 cells treated with DU145 cell derived exosomes to understand whether exosomes from AR -ve PCa cell lines influence this pathway differently. As expected, and similar to what we observed with LNCaP cell derived exosomes treatment, MEK and ERK were both phosphorylated rapidly, after 5 minutes of treatment with DU145 cell derived exosomes (Figure 8A and 8C).

### *In vivo* study in mice bearing LNCaP human tumor xenografts

**Figure 9:**
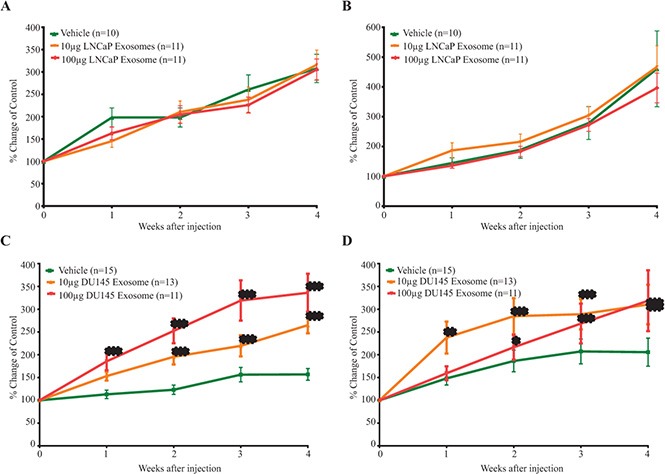
The *in vivo* effect of exosomes on the (A) tumor volume (B) PSA level of LNCaP mice xenograft treated with LNCaP exosomes and the (C) tumor volume (D) PSA level of LNCaP mice xenograft treated with DU145 exosomes Data are presented as Mean ± SEM. (***) *p* value < 0.001 was considered extremely significant compared vehicle treated-mice.

On the basis of the above findings we hypothesized that treatment of mice bearing human PCa tumour xenografts with PCa cell derived exosomes would increase the tumour volume hence promote PCa progression in a dose dependent manner. To further examine the role of PCa derived exosomes on tumor growth *in vivo*, nude mice were subcutaneously inoculated with LNCaP cells at two posterior dorsal sites. When tumor reached 100 mm^3^, mice were treated (IV tail vein) with exosomes (low, 10 μg and high, 100 μg doses) or vehicle twice/week for four weeks. Our results demonstrate no significant differences in either tumor volume or PSA level of animals treated with LNCaP exosomes (low and high dose) when compared to control (Figure 9A and 9B).

Conversely, treatment with DU145 exosomes significantly stimulated tumor growth in LNCaP xenograft bearing mice. In contrast to control mice, those treated with 100 μg of DU145 exosomes showed a very significant increase in tumor size starting after only one week of treatment (*p* value < 0.001; *n* = 11). LNCaP xenograft bearing mice treated with low dose of DU145 exosomes (10 μg) also demonstrated a very significant increase in the tumor volume after the second week of treatment with exosomes (*p* value < 0.001; Vehicle *n* = 15, 10 μg DU145 Exosome group *n* = 13 and 100 μg DU145 Exosome group *n* = 11) (Figure 9C).

As shown in Figure 9D, LNCaP xenograft bearing mice treated with DU145 exosomes demonstrate a significant increase (*p* value < 0.001, Vehicle *n* = 15, 10 μg DU145 Exosome group *n* = 13 and 100 μg DU145 Exosome group *n* = 11) in serum PSA starting one and two weeks after treatment for the high and low dose group respectively.

### Immunohistochemistry (IHC) analysis of Ki67 and filamin C expression

Upon immunohistochemical analysis of Ki67 and Filamin C expression in LNCaP tumors obtained from LNCaP tumor-bearing nude mice treated with two different concentrations of DU145 exosomes (10 μg and 100 μg), we confirmed that the expression of Filamin C had increased upon DU145 exosome treatment.

**Figure 10:**
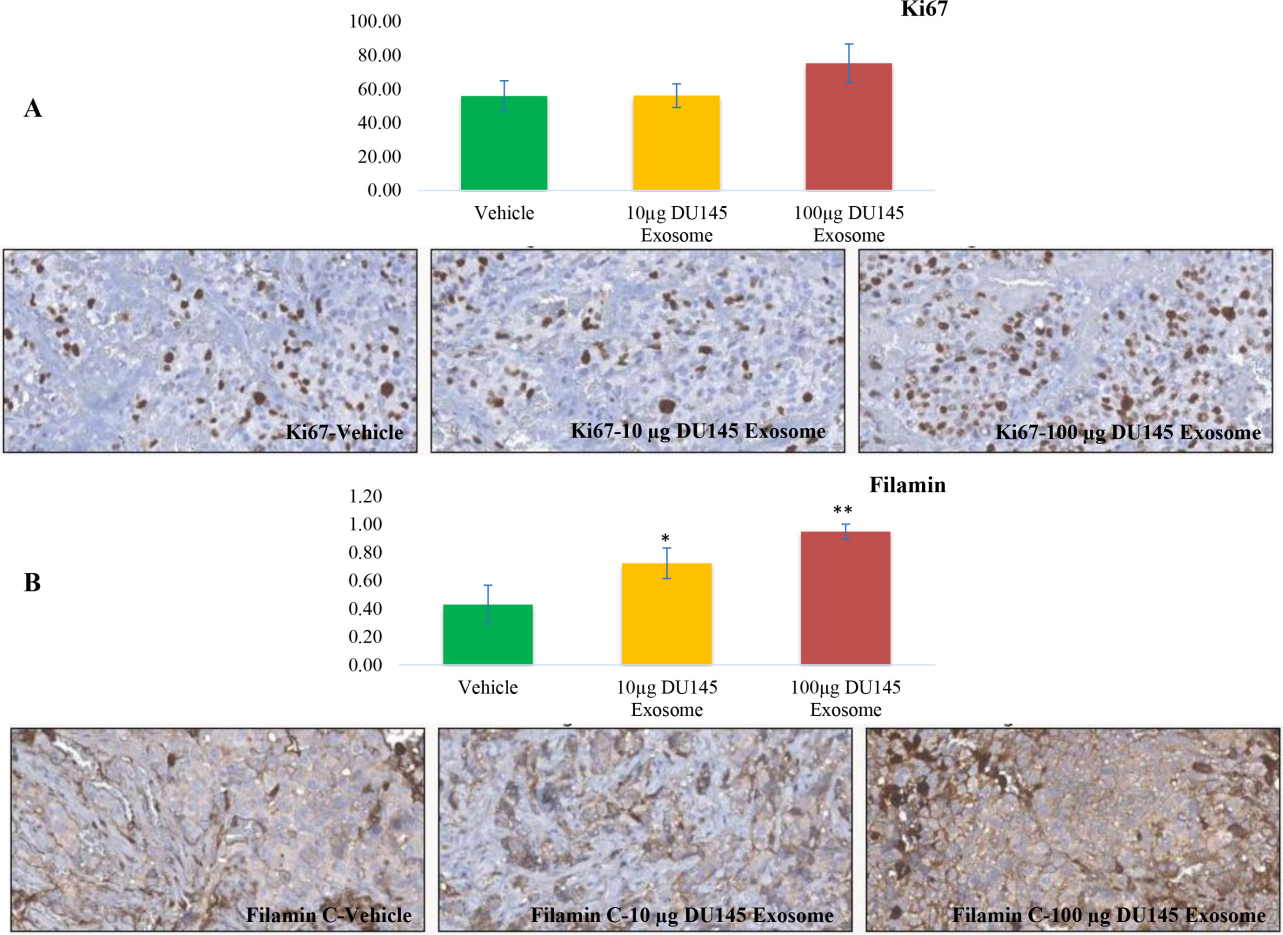
Immunohistochemical analysis Immunohistochemical analysis of (**A**) Ki67 and (**B**) Filamin C expression in LNCaP tumors upon 10 μg and 100 μg DU145 exosome treatment in comparison with vehicle treatment (*P < 0.01, **P < 0.05).

As presented in Figure 10A, the level of Ki 67 slightly increases in the LNCaP tumors treated with 100 μg of DU145 exosomes. This is in agreement with our *in vitro* proliferation results and correlates with the enhanced tumor growth profiles also observed. We have also previously reported the presence of ANXA2 (Annexin A2), CLSTN1 (Calsyntenin 1), FASN (Fatty acid Synthesis), FLNC (Filamin C, gamma), FOLH1 (Folate Hydrolase (prostate specific membrane antigen)-1) and GDF15 (Growth Differentiation Factor 15), as PCa biomarkers in exosomes derived from PCa cells [52]. Amongst these, Filamin C was one of the proteins that was specifically present in DU145 and VCaP exosomes. As revealed in Figure 10B the Filamin C level has been upregulated significantly in a dose dependent manner in LNCaP tumors upon treatment with 10 μg and 100 μg of DU145 exosomes.

## DISCUSSION

During the last decade, EVs have been proposed to be crucial players in cancer development and progression. However, the molecular mechanisms regulating the biogenesis of exosomes and their subsequent functions have only begun to be delineated. In this study, we derived a homogenous mixture of exosomes from two different PCa cell lines with different AR phenotypes and EVs isolated from both cell lines exhibited characteristic exosomal markers and lacked ER markers as validated by western blotting [52]. TEM imaging of the purified exosomes revealed the typical artificial cup-shape morphology with diameters ranging between 30–200 nm [29]. In addition to exosome characterization, the major focus of this study was to investigate and understand the *in vitro* and *in vivo* relevance of these PCa derived EVs using a series of functional assays.

Although the functional effects of EVs rely mainly on their release and internalization, the mechanistic information is very limited. Christianson *et al*. (2013) have reported that several different cellular and molecular processes/pathways could be involved in exosome uptake [61]. Their findings suggest that the heparin sulfate proteoglycan dependent entry pathway is essential for the biological activity of exosomes. However they also proposed that exosomes may employ other functional activities through alternative internalization pathways and that proteoglycan deficient cells can also attenuate exosome mediated migration as well as ERK1/2 activation [61]. Although the mechanism of exosome internalization and uptake was not the focus of this paper, it was necessary to demonstrate that all prostate cell lines in this study take up and internalize exosomes. Similar to our previous study [52], the internalization of exosomal CLU_GFP_ (derived from the LNCaP cell line) into cancer and benign prostate cell lines with different AR phenotypes was clearly demonstrated.

It is well known that the equilibrium between programmed cell death and cell survival plays a key role in the ultimate fate of cancer cells [62]. In particular, the regulation of apoptosis is known to have a central role in PCa development and it's progression to CRPC, partially due to the up-regulation of anti-apoptotic genes after androgen deprivation therapy [63–65]. Tumor derived exosomes have been shown to transport apoptosis inhibitory proteins, such as survivin, which is induced under stress conditions, in order to promote survival [66]. Yang *et al*. (2013) and Franzen *et al*. (2014) have also shown that bladder cancer cell derived exosomes inhibit tumor cell apoptosis through inhibition of the Akt and ERK pathways [67, 68]. furthermore, others have reported that cancer cell derived exosomes can create an immunosuppressive microenvironment via induction of T-cell apoptosis and this was imparted via induction of adenosine [20, 69] as well as FAS-FASL ligation [70, 71]. In agreement with what has been reported, our results indicate that PCa derived exosomes inhibit apoptosis in both cancer and benign prostate cell lines, and potentially promote tumorigenesis.

Several lines of evidence suggest that cancer derived exosomes transport paracrine signals and contribute to cancer development and progression by supporting cancer cell or endothelial cell proliferation, ultimately resulting in enhanced tumor growth [72] and angiogenesis [73]. In fact, it has been previously reported that PC3 derived EVs induce osteoclast differentiation and proliferation [74]. In this study, we demonstrate that LNCaP derived exosomes only increase the proliferation of LNCaP cells and do not influence DU145 cell proliferation, while DU145 derived exosomes increase LNCaP cell proliferation significantly but have a minimal influence on DU145 cell proliferation. These data are in agreement with the findings of Corcoran *et al*. (2012) who observed slight or no increase in both DU145 and Docetaxel Resistance DU145 cell proliferation when treated with DU145 exosomes [75]. Interestingly, the effects that we observe in benign epithelial prostate cell line (RWPE-1) exposed to PCa derived exosomes are very similar to that reported in cells of the immune system such as T-cells. At the majority of doses used, both LNCaP and DU145 derived exosomes attenuated RWPE-1 cell proliferation significantly. These positive and negative influences of PCa derived exosomes observed respectively on proliferation of PCa and benign prostate cells, supports the premise that they may play a role in tumorigenesis.

A recent bulk of accumulating evidence suggests that exosomes are key contributors to cell migration in both physiological and pathological conditions. Salomon et al. (2014) established that exosomes are released into maternal blood as early as six weeks of gestation in a normal healthy pregnancy. Furthermore, their results showed that the concentration of exosomes in maternal blood increased significantly and that these bioactive nanovesicles regulate endothelial cell migration [76]. On the other hand, growing evidence has also shown that cancer-derived exosomes can play a central role in various aspects of cancer progression via activation of different pathways. Activation of the Wnt-planar cell polarity signaling pathway in breast cancer cells as a result of treatment with fibroblast-secreted exosomes as well as secretion of HSP90α shown to be promoted by breast cancer exosomes or EDIL-3 via bladder cancer exosomes are just few examples of how exosomes can influence cell migration and invasion in cancer models [77–79].

In addition, Bijnsdorp *et al*. (2013) and Morello *et al*. (2013) have clearly demonstrated that PCa derived exosomes or large oncosomes increase the migration and invasion of noncancerous and cancer associated fibroblasts [80, 81]. In agreement with this, our three independent migration assays demonstrate that both AR+/−ve PCa derived exosomes significantly promote cell migration, motility and metastasis. Specifically, our results reveal that DU145 derived exosomes promote the migration of RWPE-1 and DU145 cells to a greater extent than LNCaP derived exosomes. However, LNCaP cell derived exosomes appear to have a greater effect on 3D migration compared to DU145 derived exosomes.

As discussed, our results support the premise that PCa derived exosomes (with two different AR phenotype) are important players in PCa progression due to their role in reducing apoptosis and inducing proliferation, migration and invasion. A growing amount of EV research describes the fact that exosomes can promote cancer cell proliferation, migration and invasion via activation of the MAPK signalling pathway [82]. Induction of the MAPK pathway has been shown to be correlate with tumor grade, stage and PCa progression [60] and consequently, the inhibition of this pathway has also been the subject of intense scrutiny and pharmacological research for cancer treatment [23, 83–87]. FASL^+^ exosomes have been shown to activate c-FLIP_L,_ ERK and NF-κB pathways and therefore increase MMP expression in tumor cells leading to tumor invasion [88]. Ye *et al*. (2014) have also demonstrated that nasopharyngeal carcinoma derived exosomes mediate T-cell dysfunction such as proliferation, differentiation and cytokine secretion via down-regulation of MAPK1 and JAK/STAT pathways [89]. In agreement with these observations our results support the premise that PCa derived exosomes activate the MEK/ERK pathway in both cancer and benign PCa cells. In all but C4-2 cells, we demonstrated that MEK or ERK phosphorylation was enhanced upon exosome treatment. While the activation of MAPK could somewhat explain the effect of exosomes on PCa cells, the molecular and cellular mechanisms involved still remain to be thoroughly elucidated.

In agreement with our *in vitro* observations we also showed that DU145 derived exosomes influence tumour development and PSA induction in LNCaP xenograft mouse model. The significant induction of Filamin C upon treatment with DU145 exosomes observed in LNCaP xenograft bearing mice, provides further evidence to support the role of exosomes in cancer progression and also infer the selective uptake of PCa derived exosomes *in vivo* by the PCa xenograft tumor.

In conclusion, this study truly sheds light on the important role of PCa derived exosomes in PCa development and progression. Taken together, all the data presented and discussed herein demonstrates the significance of exosomal influence on several functional processes in PCa, corroborating the likelihood that PCa derived exosomes play a pivotal role in PCa development and progression.

## MATERIALS AND METHODS

### Cell culture

PC3 and DU145 human prostate cancer cells (ATCC) were grown in Dulbecco's Modified Eagle's Medium (DMEM), LNCaP cells (ATCC) were cultured in RPMI 1640 supplemented with 5% FBS (Invitrogen) and antibiotic, at 37°C in 5% CO_2_. RWPE-1 (ATCC) cells were maintained in keratinocyte-SFM (KSFM) with growth supplement (GIBCO) and 1% penicillin streptomycin (Invitrogen, Carlsbad, CA). CLU_GFP_ stably over-expressing LNCaP cells were maintained in 200 mg/ml G418 (Invitrogen) containing RPMI supplemented with 10% FBS and 1% antibiotics at 37°C in 5% CO_2_.

All cells were grown to 60–70% confluency, washed with sterile PBS buffer and removed from serum and incubated in culture media for 72 h for exosome collection and purification.

### Exosome isolation

Exosomes were purified from the media of AR +ve and –ve PCa cell lines following exposure to LNCaP and DU145 cells for 72 hours. For exosome purification, 200 ml of each cell line's conditioned medium was precleared by centrifugation at 6,000 rpm at 4°C for 10 minutes to remove cell debris and protein aggregates. The precleared medium was concentrated to 2 ml using a 100 kDa MWCO Centricon Plus-20 filter capsule (Millipore, Billerica, MA). Samples were transferred to ultracentrifuge tubes containing 300 μl of 30% sucrose-deuterium oxide (D_2_O). Sample tubes were then ultracentrifuged at 100,000 g for 70 minutes at 4°C. Purified exosomes (350 μl) were collected from the cushion of sucrose and washed with PBS prior to any exosome treatment.

### Transmission electron microscopy (TEM)

Isolated exosomes (2.5 μl) were dried onto freshly ‘glow discharged’ 300 mesh formvar/carbon-coated TEM grids (Ted Pella, Redding, CA), negatively stained with 2% aqueous uracyl acetate and observed under a Hitachi H7600 TEM (Hitachi High-Technologies Corp., Tokyo, Japan) operated at 80kV. Images were captured with a side mounted 1K AMT Advantage digital camera (Advanced Microscopy Techniques, Corp. Woburn, MA).

### Western blot analysis

Exosomes and cell lysates were analyzed for total protein concentration using the BCA protein determination kit (Sigma, Oakville, Ontario, Canada). Thirty micrograms of total protein associated with purified exosomes and their corresponding cell lysate were loaded on 12% acrylamaide gel. Relative enzyme levels were detected using antibodies specific for exosome markers: mouse monoclonal Actin (1:1000 Sigma) mouse monoclonal Alix and mouse monoclonal HSP70 (1:1000 Santa Cruz Biotechnology, Inc., Santa Cruz, CA). In order to evaluate the purity of the exosome preparations, all exosomes samples were also blotted against GRP94 (1:1000 Cell Signaling) to demonstrate the absence of cellular contaminants from cell lysate in the exosome preparation. The activation of MEK/ERK pathway was demonstrated using rabbit polyclonal p-MEK1/2, t-MEK1/2, p-ERK1/2, and t-ERK1/2 antibodies (1:1000 Cell signalling).

### Nanoparticle tracking analysis (NTA)

Size distribution and the estimated concentration of nanoparticles in each purified exosome isolate were analysed using a light scattering technology via measurement of the rate of Brownian motion with the NanoSight™ LM10 system (NanoSight Ltd, Amesbury, UK) configured with a 488 nm laser and a high sensitivity digital camera (OrcaFlash2.8, Hamamatsu C11440, NanoSight Ltd).

All samples were diluted with nanoparticle-free water to obtain exosome concentration within the range of 5 × 10^7^ to 5 × 10^9^ particle/ml. Samples were administered and recorded under controlled flow (infusion rate of 100) using a NanoSight™ syringe pump and script control system. The ambient temperature was set at 25°C, with the camera sensitivity and detection threshold set between 9 to 12 for maximum particle detection. Five different videos of 60 seconds from 3 different replicates were collected and analysed using NTA-software (version 2.3) for each sample.

### Confocal microscopy

In order to study the uptake of exosomes by different cancerous or non-cancerous prostate cell lines (with distinct AR expression phenotypes), equal numbers of cells were seeded in four-well chamber slides (Lab-Tek II chamber slide with cover, Thermo Fisher scientific). Next, as previously reported [52] fresh CLU_GFP_ labelled exosomes were incubated with PC3 (AR−ve) and LNCaP (AR+ve) PCa cell lines as well as RWPE-1 representing a benign epithelial prostate cell line, for 12 h at 37°C and 5% CO_2_. CLU_GFP_ tagged exosomes were isolated from a CLU_GFP_ stably overexpressing LNCaP cell line. After removal of media, cells were fixed with ice-cold MeOH/Acetone (3:1) for 10 minutes, and then washed in TBS buffer and permeabilized in 0.1% Triton X-100 in TBS for 15 minutes at room temperature (RT). Non-specific binding was avoided by blocking in odyssey solution for 30 minutes at RT. Primary purified mouse anti E-Cadherin (1:250 BD Transduction Laboratories™) or rabbit anti Caveolin-1 (1:250 Santa Cruz, CA) were diluted in blocking agent and incubated with cells for 1 hour at RT. Secondary antibody, Alexa Fluor^®^ 568 goat antimouse IgG or Alexa Fluor^®^ 555 Donkey Anti-Rabbit IgG (1:500, Invitrogen), was incubated with cells for 30 minutes at RT. Finally, as described above, all slide chambers were mounted and monitored using confocal microscopy (LSM 780 Ziess, Heidelberg, Germany).

### Apoptosis assay

Caspase-3/7 assay was carried out by mixing 10 μg of total protein extracts prepared from cells as above with Caspase-Glo 3/7 substrates (Promega) [90]. The relative luminescence units (RLU) were measured using a Synergy H4 Hybrid Multi-Mode Microplate Reader (BioTek Instruments, Inc.). The percentage of apoptosis based on caspase 3/7 activity was calculated relative to that of control samples.

### Real time cell analysis (xCELLigence)

#### Proliferation

A blank reading was taken with 30 μl of RPMI + 5% FBS in each well. DU145 cells were seeded at a density of 7,000 cells per well, LNCaP and RWPE-1 cells were seeded at a density of 20,000 cells per well with a final volume of 100 μl. Treatments of either DU145 or LNCaP exosomes (0–400 μg/ml) were added after 24 hours to a final volume of 200 μl. Impedance measurement were recorded once every 5 minutes for the first 25 sweeps, followed by once every 10 minutes till completion of the experiment 72 hours post treatment. Data was normalized to the last time point prior to treatment delivery.

#### Migration

Media with 10% FBS was added to the bottom chamber and 30 μl of serum free media was added to the wells of the top chamber of the CIM migration plates (Post equilibration of the two chambers). DU145 and RWPE-1 cells were seeded at a density of 20,000 cells per well with a final volume of 100 μl. Treatments of either DU145 or LNCaP exosomes (100 μg/ml) was added after 24 hours to a final volume of 200 μl. Impedance measurement were recorded once every 5 minutes for the first 25 sweeps, followed by once every 10 minutes till completion of the experiment 48 hours post treatment. Data was normalized to the last time point prior to treatment delivery.

#### Cell motility

*In vitro* cell migration assays were performed in a 24-well Transwell plate with 8-μm polycarbonate membrane filters (Corning) separating the lower and upper culture chambers. RWPE-1 cells were grown to subconfluence (~75%–80%) and were incubated with LNCaP or DU145-derived exosomes (100 μg/ml) or serum-free medium for 48 hours. After detachment with trypsin, cells were washed with PBS and resuspended in serum-free medium, after which the cell suspension (1 × 10^5^ cells), supplemented with exosomes (100 μg/ml) or serum-free medium, was added to the upper chamber. Medium containing 10% FBS and exosomes (100 μg/ml) or serum-free medium was added to the bottom wells of the chamber. The cells that had not migrated were removed from the upper face of the filters using cotton swabs, and the cells that had migrated to the lower face of the filters were fixed with methanol and stained with 0.5% crystal violet solution. Images of at least 10 random fields were captured from each membrane using a ×10 objective, and the number of migratory cells was counted. All values are representative of at least two independent experiments.

#### 3D migration

Three-dimensional multicellular spheroids were prepared by the liquid overlay technique [91]. In brief, tissue culture microplates were coated with 75 μl of 1% agarose in water. RWPE-1 cells grown as a monolayer were resuspended with trypsin, and 2 × 10^3^ cells were seeded in microwells so as to obtain a single spheroid per well after 3 days.

Serum-free medium or serum-free medium supplemented with LNCaP or DU145 exosomes (final concentration, 0.1 μg/μl) was added to the microwells containing spheroids.

The spheroid-based assay we performed 48 hours later, [57]. Briefly, RWPE-1 spheroids were transferred onto a 50 μg/ml collagen I-coated surface (single spheroid/96-well; 6 spheroids/treatment) in 300 μl of media in the presence or absence of LNCaP or DU145 exosomes (final concentration of 0.1 μg/μl). The spheroids were imaged (Canon EOS Digital) and the migration was quantified by recording the total area covered by RWPE-1 cells at the start of the experiment and at 12 and 24 hours post treatment. The covered areas were manually measured using ImageJ and the data have been normalized to the original size of each spheroid recorded at *t* = 0 (formula: (migrated area at *t* = *x*/migrated area at *t* = 0) × 100).

*In vivo* Study in Mice bearing LNCaP Human Tumor Xenografts

Animal studies were carried out according to the guidelines of the Animal Ethics Committee at the University of British Columbia (UBC). The approved UBC Animal Ethics protocol, granted to Dr. Emma Tomlinson Guns' lab for this work, was #A11-0337. Six to eight week-old nude mice (Harlan Sprague Dawley, Inc.) weighing 25-31 grams were subcutaneously inoculated with LNCaP cells (10^6^ cells in BD matrigel, BD Biosciences, New Jersey, USA) at the right posterior dorsal site. Body weight, tumor volume and serum PSA levels were measured weekly. When the tumor volume reached 100 mm^3^, mice were randomized into 3 treatment groups; vehicle, low dose (10 μg protein of LNCaP or DU145 derived exosomes) and high dose (100 μg LNCaP or DU145 derived Exosomes) treatment. All mice were injeced intravenously via the tail vein twice a week for 4 weeks. Calipers were used to measure the three perpendicular axes of each tumor.

$$V=(L\times W\times H)\frac{\pi }{6}$$

The above formula, where L is the length, W the width, and H the height, was used to calculate the tumor volume. Mice were also weighed weekly and monitored daily for signs of toxicity including death, lethargy, blindness and disorientation.

#### Immunohistochemistry (IHC)

IHC was carried out for a total of 19 xenograft tumors from LNCaP cells. The H & E slides were reviewed and the desired areas were marked on them and their correspondent paraffin blocks. TMA was manually constructed (Beecher Instruments, MD, USA) by punching multiple cores of 1 mm for each sample. All the specimen were from xenograft tumors.

Immunohistochemical staining was conducted by Ventana autostainer model Discover XT^™^ (Ventana Medical System, Tuscan, Arizona) with enzyme labeled biotin streptavidin system and solvent resistant Red Map kit by using 1:500 of ki67 rabbit polyclonal antibody (Thermoscientific), 1:2,000 concentrations of Filamin mouse monoclonal antibody (abcam), 1:50 of GDF15 Rabbit polyclonal antibody (Abcam) and 1:200 of Caveolin-1 rabbit polyclonal antibody (Cell Signaling).

#### Scoring system

Values on a four-point scale were assigned to each immunostain of Filamin, GDF15 and Caveolin-1. Descriptively, 0 represents no staining by any tumor cells, 1 represents a faint or focal, questionably present stain, 2 represents a stain of convincing intensity in a minority of cells and 3 a stain of convincing intensity in a majority of cells.

Proliferation factor was scored by calculating of the average of cell counts of 3 HPF in each core.

#### Statistical analysis

Unless indicated, analyses were performed on data generated from triplicate experiments. Results were expressed as mean ± standard deviation. For most experiments, unless indicated, statistical significance for differences were evaluated by student *t*-test (*P* < 0.05).

In Real Time Cell Analysis (xCELLigence) experiments student *t*-test Level of significance was set at *P < 0.05, **P < 0.01, ***P < 0.001 and the slope was calculated by using the RTCA 2.0 software (ACEA).

## Abbreviations

USA: United States of America
AR: Androgen Receptor
Akt: Protein kinase B
CD3: Cluster of Differentiation
CLU: Clusterin
CRPC: Castration Resistant Prostate Cancer
D^2^O: Deuterium Oxide
DMEM: Dulbecco’s Modified Eagle’s Medium
DNA: Deoxyribonucleic acid
EE: Early Endosome
ER: Endoplasmic Reticulum
ERK: Extracellular Signal-Regulated Kinases
ESCRT: Endosomal Sorting Complexes Required for Transport
EV: Extracellular Vesicles
FBS: Fetal Bovine Serum
FOLH1: Folate Hydrolase
FP: Green Fluorescent Protein
HSP: Heat Shock Protein
MAPK: Mitogen-Activated Protein Kinases
MEK: Mitogen-Activated Protein Kinase Kinases
MeOH: Methanol
MHC: Major Histocompatibility Complex
MVE: Multivesicular Endosome
NTA: Nanoparticle Tracking Analysis
PCa: Prostate Cancer
PI3K: Phosphoinositide 3-kinase
PSA: Prostate Specific Antigen
RNA: Ribonucleic Acid
RPMI: Roswell Park Memorial Institute
RT: Room Temperature
TEM: Transmission Electron Microscopy

## References

[1] Shen MM, Abate-Shen C (2010). Molecular genetics of prostate cancer: new prospects for old challenges. Genes Dev.

[2] Jemal A, Siegel R, Xu J, Ward E (2010). Cancer statistics, 2010. Cancer J Clin.

[3] DeSantis CE, Lin CC, Mariotto AB, Siegel RL, Stein KD, Kramer JL, Alteri R, Robbins AS, Jemal A (2014). Cancer treatment and survivorship statistics, 2014. Cancer J Clin.

[4] Garzotto M, Hung AY (2010). Contemporary management of high-risk localized prostate cancer. Curr Urol reports.

[5] Miyake H, Nelson C, Rennie PS, Gleave ME (2000). Overexpression of insulin-like growth factor binding protein-5 helps accelerate progression to androgen-independence in the human prostate LNCaP tumor model through activation of phosphatidylinositol 3′-kinase pathway. Endocrinology.

[6] Knudsen KE, Scher HI (2009). Starving the addiction:new opportunities for durable suppression of AR signaling in prostate cancer. Clin Cancer Res.

[7] Craft N, Shostak Y, Carey M, Sawyers CL (1999). A mechanism for hormone-independent prostate cancer through modulation of androgen receptor signaling by the HER-2/neu tyrosine kinase. Nature Med.

[8] Culig Z (2004). Androgen receptor cross-talk with cell signalling pathways. Growth Factors.

[9] Petrylak DP, Tangen CM, Hussain MH, Lara PN, Jones JA, Taplin ME, Burch PA, Berry D, Moinpour C, Kohli M, Benson MC, Small EJ (2004). Docetaxel and estramustine compared with mitoxantrone and prednisone for advanced refractory prostate cancer. N Engl J Med.

[10] Tannock IF, de Wit R, Berry WR, Horti J, Pluzanska A, Chi KN, Oudard S, Theodore C, James ND, Turesson I, Rosenthal MA, Eisenberger MA, TAX 327 Investigators (2004). Docetaxel plus prednisone or mitoxantrone plus prednisone for advanced prostate cancer. N Engl J Med.

[11] Cookson MS, Roth BJ, Dahm P, Engstrom C, Freedland SJ, Hussain M, Lin DW, Lowrance WT, Murad MH, Oh WK, Penson DF, Kibel AS (2013). Castration-resistant prostate cancer: AUA Guideline. J Urol.

[12] Al-Nedawi K, Meehan B, Micallef J, Lhotak V, May L, Guha A, Rak J (2008). Intercellular transfer of the oncogenic receptor EGFRvIII by microvesicles derived from tumour cells. Nature Cell Biol.

[13] Anderson HC (1969). Vesicles associated with calcification in the matrix of epiphyseal cartilage. J Cell Biol.

[14] Muralidharan-Chari V, Clancy JW, Sedgwick A, D'Souza-Schorey C (2010). Microvesicles: mediators of extracellular communication during cancer progression. J Cell Sci.

[15] Ratajczak J, Miekus K, Kucia M, Zhang J, Reca R, Dvorak P, Ratajczak MZ (2006). Embryonic stem cell-derived microvesicles reprogram hematopoietic progenitors: evidence for horizontal transfer of mRNA and protein delivery. Leukemia.

[16] Yang M, Chen J, Su F, Yu B, Su F, Lin L, Liu Y, Huang JD, Song E (2011). Microvesicles secreted by macrophages shuttle invasion-potentiating microRNAs into breast cancer cells. Mol Cancer.

[17] Cocucci E, Racchetti G, Meldolesi J (2009). Shedding microvesicles: artefacts no more. Trends Cell Biol.

[18] Shedden K, Xie XT, Chandaroy P, Chang YT, Rosania GR (2003). Expulsion of small molecules in vesicles shed by cancer cells: association with gene expression and chemosensitivity profiles. Cancer Res.

[19] Taylor DD, Gercel-Taylor C (2005). Tumour-derived exosomes and their role in cancer-associated T-cell signalling defects. Br J Cancer.

[20] Liu C, Yu S, Zinn K, Wang J, Zhang L, Jia Y, Kappes JC, Barnes S, Kimberly RP, Grizzle WE, Zhang HG (2006). Murine mammary carcinoma exosomes promote tumor growth by suppression of NK cell function. J Immunol.

[21] Abusamra AJ, Zhong Z, Zheng X, Li M, Ichim TE, Chin JL, Min WP (2005). Tumor exosomes expressing Fas ligand mediate CD8+ T-cell apoptosis. Blood Cells Mol Dis.

[22] Koga K, Matsumoto K, Akiyoshi T, Kubo M, Yamanaka N, Tasaki A, Nakashima H, Nakamura M, Kuroki S, Tanaka M, Katano M (2005). Purification, characterization and biological significance of tumor-derived exosomes. Anticancer Res.

[23] Qu JL, Qu XJ, Zhao MF, Teng YE, Zhang Y, Hou KZ, Jiang YH, Yang XH, Liu YP (2009). Gastric cancer exosomes promote tumour cell proliferation through PI3K/Akt and MAPK/ERK activation. Digestive Liver Dis.

[24] Thery C, Zitvogel L, Amigorena S (2002). Exosomes: composition, biogenesis and function. Nature Rev Immunol.

[25] Aharon A, Brenner B (2009). Microparticles, thrombosis and cancer. Best practice & research. Clin Haematol.

[26] Thery C, Ostrowski M, Segura E (2009). Membrane vesicles as conveyors of immune responses. Nature Rev Immunol.

[27] Colombo M, Raposo G, Thery C (2014). Biogenesis, secretion, and intercellular interactions of exosomes and other extracellular vesicles. Annu Rev Cell Dev Biol.

[28] Parolini I, Federici C, Raggi C, Lugini L, Palleschi S, De Milito A, Coscia C, Iessi E, Logozzi M, Molinari A, Colone M, Tatti M, Sargiacomo M (2009). Microenvironmental pH is a key factor for exosome traffic in tumor cells. J Biol Chem.

[29] Katakowski M, Buller B, Zheng X, Lu Y, Rogers T, Osobamiro O, Shu W, Jiang F, Chopp M (2013). Exosomes from marrow stromal cells expressing miR-146b inhibit glioma growth. Cancer Letters.

[30] Camussi G, Deregibus MC, Bruno S, Grange C, Fonsato V, Tetta C (2011). Exosome/microvesicle-mediated epigenetic reprogramming of cells. Am J Cancer Res.

[31] Fabbri M, Paone A, Calore F, Galli R, Gaudio E, Santhanam R, Lovat F, Fadda P, Mao C, Nuovo GJ, Zanesi N, Crawford M, Ozer GH (2012). MicroRNAs bind to Toll-like receptors to induce prometastatic inflammatory response. Proc Nat Acad Sci USA.

[32] Caby MP, Lankar D, Vincendeau-Scherrer C, Raposo G, Bonnerot C (2005). Exosomal-like vesicles are present in human blood plasma. Int Immunol.

[33] Taylor DD, Akyol S, Gercel-Taylor C (2006). Pregnancy-associated exosomes and their modulation of T cell signaling. J Immunol.

[34] Taylor DD, Gercel-Taylor C (2008). MicroRNA signatures of tumor-derived exosomes as diagnostic biomarkers of ovarian cancer. Gynecol Oncol.

[35] Andre F, Schartz NE, Movassagh M, Flament C, Pautier P, Morice P, Pomel C, Lhomme C, Escudier B, Le Chevalier T, Tursz T, Amigorena S, Raposo G (2002). Malignant effusions and immunogenic tumour-derived exosomes. Lancet.

[36] Bard MP, Hegmans JP, Hemmes A, Luider TM, Willemsen R, Severijnen LA, van Meerbeeck JP, Burgers SA, Hoogsteden HC, Lambrecht BN (2004). Proteomic analysis of exosomes isolated from human malignant pleural effusions. Am. J. Respir. Cell Mol Biol.

[37] Pisitkun T, Shen RF, Knepper MA (2004). Identification and proteomic profiling of exosomes in human urine. Proc Nat Acad Sci USA.

[38] Asea A, Jean-Pierre C, Kaur P, Rao P, Linhares IM, Skupski D, Witkin SS (2008). Heat shock protein-containing exosomes in mid-trimester amniotic fluids. J Reprod Immunol.

[39] Admyre C, Grunewald J, Thyberg J, Gripenback S, Tornling G, Eklund A, Scheynius A, Gabrielsson S (2003). Exosomes with major histocompatibility complex class II and co-stimulatory molecules are present in human BAL fluid. Eur Respir J.

[40] Hawari FI, Rouhani FN, Cui X, Yu ZX, Buckley C, Kaler M, Levine SJ (2004). Release of full-length 55-kDa TNF receptor 1 in exosome-like vesicles: a mechanism for generation of soluble cytokine receptors. Proc Nat Acad Sci USA.

[41] Admyre C, Johansson SM, Qazi KR, Filen JJ, Lahesmaa R, Norman M, Neve EP, Scheynius A, Gabrielsson S (2007). Exosomes with immune modulatory features are present in human breast milk. J Immunol.

[42] Thery C, Boussac M, Veron P, Ricciardi-Castagnoli P, Raposo G, Garin J, Amigorena S (2001). Proteomic analysis of dendritic cell-derived exosomes: a secreted subcellular compartment distinct from apoptotic vesicles. J Immunol.

[43] van der Pol E, Boing AN, Harrison P, Sturk A, Nieuwland R (2012). Classification, functions, and clinical relevance of extracellular vesicles. Pharmacol. Rev.

[44] Thakur BK, Zhang H, Becker A, Matei I, Huang Y, Costa-Silva B, Zheng Y, Hoshino A, Brazier H, Xiang J, Williams C, Rodriguez-Barrueco R, Silva JM (2014). Double-stranded DNA in exosomes: a novel biomarker in cancer detection. Cell Res.

[45] Kahlert C, Melo SA, Protopopov A, Tang J, Seth S, Koch M, Zhang J, Weitz J, Chin L, Futreal A, Kalluri R (2014). Identification of double-stranded genomic DNA spanning all chromosomes with mutated KRAS and p53 DNA in the serum exosomes of patients with pancreatic cancer. J Biol Chem.

[46] Lotvall J, Valadi H (2007). Cell to cell signalling via exosomes through esRNA. Cell Adh Migr.

[47] Huang X, Yuan T, Tschannen M, Sun Z, Jacob H, Du M, Liang M, Dittmar RL, Liu Y, Liang M, Kohli M, Thibodeau SN, Boardman L (2013). Characterization of human plasma-derived exosomal RNAs by deep sequencing. BMC Genomics.

[48] De Smaele E, Ferretti E, Gulino A (2010). MicroRNAs as biomarkers for CNS cancer and other disorders. Brain Res.

[49] Stoorvogel W, Kleijmeer MJ, Geuze HJ, Raposo G (2002). The biogenesis and functions of exosomes. Traffic.

[50] Ronquist KG, Ronquist G, Larsson A, Carlsson L (2010). Proteomic analysis of prostate cancer metastasis-derived prostasomes. Anticancer Res.

[51] Arcaro A, Guerreiro AS (2007). The phosphoinositide 3-kinase pathway in human cancer: genetic alterations and therapeutic implications. Curr Genomics.

[52] Hosseini-Beheshti E, Pham S, Adomat H, Li N, Tomlinson Guns ES (2012). Exosomes as biomarker enriched microvesicles: characterization of exosomal proteins derived from a panel of prostate cell lines with distinct AR phenotypes. Mol. & Cell. Proteomics.

[53] Gercel-Taylor C, Atay S, Tullis RH, Kesimer M, Taylor DD (2012). Nanoparticle analysis of circulating cell-derived vesicles in ovarian cancer patients. Anal Biochem.

[54] Sarker S, Scholz-Romero K, Perez A, Illanes SE, Mitchell MD, Rice GE, Salomon C (2014). Placenta-derived exosomes continuously increase in maternal circulation over the first trimester of pregnancy. J Transl Med.

[55] Roshan Moniri M, Young A, Reinheimer K, Rayat J, Dai LJ, Warnock GL (2015). Dynamic assessment of cell viability, proliferation and migration using real time cell analyzer system (RTCA). Cytotechnology.

[56] Smalley KS, Lioni M, Herlyn M (2006). Life isn't flat: taking cancer biology to the next dimension. *In vitro* cellular & developmental biology. Animal.

[57] Vinci M, Box C, Zimmermann M, Eccles SA (2013). Tumor spheroid-based migration assays for evaluation of therapeutic agents. Methods Mol Biol.

[58] Mukherjee R, Bartlett JM, Krishna NS, Underwood MA, Edwards J (2005). Raf-1 expression may influence progression to androgen insensitive prostate cancer. Prostate.

[59] Weber MJ, Gioeli D (2004). Ras signaling in prostate cancer progression. J Cell Biochem.

[60] Gioeli D, Mandell JW, Petroni GR, Frierson HF (1999). Weber MJ. Activation of mitogen-activated protein kinase associated with prostate cancer progression. Cancer Res.

[61] Christianson HC, Svensson KJ, van Kuppevelt TH, Li JP, Belting M (2013). Cancer cell exosomes depend on cell-surface heparan sulfate proteoglycans for their internalization and functional activity. Proc Nat Acad Sci USA.

[62] Ouyang L, Shi Z, Zhao S, Wang FT, Zhou TT, Liu B, Bao JK (2012). Programmed cell death pathways in cancer: a review of apoptosis, autophagy and programmed necrosis. Cell Proliferat.

[63] Li Y, Che M, Bhagat S, Ellis KL, Kucuk O, Doerge DR, Abrams J, Cher ML, Sarkar FH (2004). Regulation of gene expression and inhibition of experimental prostate cancer bone metastasis by dietary genistein. Neoplasia.

[64] Zhang M, Latham DE, Delaney MA, Chakravarti A (2005). Survivin mediates resistance to antiandrogen therapy in prostate cancer. Oncogene.

[65] Guo Z, Dai B, Jiang T, Xu K, Xie Y, Kim O, Nesheiwat I, Kong X, Melamed J, Handratta VD, Njar VC, Brodie AM, Yu LR (2006). Regulation of androgen receptor activity by tyrosine phosphorylation. Cancer Cell.

[66] Khan S, Jutzy JM, Valenzuela MM, Turay D, Aspe JR, Ashok A, Mirshahidi S, Mercola D, Lilly MB, Wall NR (2012). Plasma-derived exosomal survivin, a plausible biomarker for early detection of prostate cancer. PloS One.

[67] Yang L, Wu XH, Wang D, Luo CL, Chen LX (2013). Bladder cancer cell-derived exosomes inhibit tumor cell apoptosis and induce cell proliferation *in vitro*. Mol Med Reports.

[68] Franzen CA, Simms PE, Van Huis AF, Foreman KE, Kuo PC, Gupta GN (2014). Characterization of uptake and internalization of exosomes by bladder cancer cells. BioMed Res Int.

[69] Clayton A, Al-Taei S, Webber J, Mason MD, Tabi Z (2011). Cancer exosomes express CD39 and CD73, which suppress T cells through adenosine production. J Immunol.

[70] Huber V, Fais S, Iero M, Lugini L, Canese P, Squarcina P, Zaccheddu A, Colone M, Arancia G, Gentile M, Seregni E, Valenti R, Ballabio G (2005). Human colorectal cancer cells induce T-cell death through release of proapoptotic microvesicles: role in immune escape. Gastroenterology.

[71] Andreola G, Rivoltini L, Castelli C, Huber V, Perego P, Deho P, Squarcina P, Accornero P, Lozupone F, Lugini L, Stringaro A, Molinari A, Arancia G (2002). Induction of lymphocyte apoptosis by tumor cell secretion of FasL-bearing microvesicles. J Exp Med.

[72] Peinado H, Aleckovic M, Lavotshkin S, Matei I, Costa-Silva B, Moreno-Bueno G, Hergueta-Redondo M, Williams C, Garcia-Santos G, Ghajar C, Nitadori-Hoshino A, Hoffman C, Badal K (2012). Melanoma exosomes educate bone marrow progenitor cells toward a pro-metastatic phenotype through MET. Nature Med.

[73] Umezu T, Tadokoro H, Azuma K, Yoshizawa S, Ohyashiki K, Ohyashiki JH (2014). Exosomal miR-135b shed from hypoxic multiple myeloma cells enhances angiogenesis by targeting factor-inhibiting HIF-1. Blood.

[74] Inder KL, Ruelcke JE, Petelin L, Moon H, Choi E, Rae J, Blumenthal A, Hutmacher D, Saunders NA, Stow JL, Parton RG, Hill MM (2014). Cavin-1/PTRF alters prostate cancer cell-derived extracellular vesicle content and internalization to attenuate extracellular vesicle-mediated osteoclastogenesis and osteoblast proliferation. J. Extracell. Vesicles.

[75] Corcoran C, Rani S, O'Brien K, O'Neill A, Prencipe M, Sheikh R, Webb G, McDermott R, Watson W, Crown J, O'Driscoll L (2012). Docetaxel-resistance in prostate cancer: evaluating associated phenotypic changes and potential for resistance transfer via exosomes. PloS One.

[76] Salomon C, Torres MJ, Kobayashi M, Scholz-Romero K, Sobrevia L, Dobierzewska A, Illanes SE, Mitchell MD, Rice GE (2014). A gestational profile of placental exosomes in maternal plasma and their effects on endothelial cell migration. PloS One.

[77] Luga V, Zhang L, Viloria-Petit AM, Ogunjimi AA, Inanlou MR, Chiu E, Buchanan M, Hosein AN, Basik M, Wrana JL (2012). Exosomes mediate stromal mobilization of autocrine Wnt-PCP signaling in breast cancer cell migration. Cell.

[78] McCready J, Sims JD, Chan D, Jay DG (2010). Secretion of extracellular hsp90alpha via exosomes increases cancer cell motility: a role for plasminogen activation. BMC Cancer.

[79] Beckham CJ, Olsen J, Yin PN, Wu CH, Ting HJ, Hagen FK, Scosyrev E, Messing EM, Lee YF (2014). Bladder cancer exosomes contain EDIL-3/Del1 and facilitate cancer progression. J Urol.

[80] Bijnsdorp IV, Geldof AA, Lavaei M, Piersma SR, van Moorselaar RJ, Jimenez CR (2013). Exosomal ITGA3 interferes with non-cancerous prostate cell functions and is increased in urine exosomes of metastatic prostate cancer patients. J Extracell Vesicles.

[81] Morello M, Minciacchi VR, de Candia P, Yang J, Posadas E, Kim H, Griffiths D, Bhowmick N, Chung LW, Gandellini P, Freeman MR, Demichelis F, Di Vizio D (2013). Large oncosomes mediate intercellular transfer of functional microRNA. Cell Cycle.

[82] Morgan K, Stavrou E, Leighton SP, Miller N, Sellar R, Millar RP (2011). Elevated GnRH receptor expression plus GnRH agonist treatment inhibits the growth of a subset of papillomavirus 18-immortalized human prostate cells. Prostate.

[83] Roberts PJ, Der CJ (2007). Targeting the Raf-MEK-ERK mitogen-activated protein kinase cascade for the treatment of cancer. Oncogene.

[84] Meckes DG, Shair KH, Marquitz AR, Kung CP, Edwards RH, Raab-Traub N (2010). Human tumor virus utilizes exosomes for intercellular communication. Proc Nat Acad USA.

[85] Sirois I, Raymond MA, Brassard N, Cailhier JF, Fedjaev M, Hamelin K, Londono I, Bendayan M, Pshezhetsky AV, Hebert MJ (2011). Caspase-3-dependent export of TCTP: a novel pathway for antiapoptotic intercellular communication. Cell Death Differ.

[86] Chalmin F, Ladoire S, Mignot G, Vincent J, Bruchard M, Remy-Martin JP, Boireau W, Rouleau A, Simon B, Lanneau D, De Thonel A, Multhoff G, Hamman A (2010). Membrane-associated Hsp72 from tumor-derived exosomes mediates STAT3-dependent immunosuppressive function of mouse and human myeloid-derived suppressor cells. J Clin Invest.

[87] Deleault KM, Skinner SJ, Brooks SA (2008). Tristetraprolin regulates TNF TNF-alpha mRNA stability via a proteasome dependent mechanism involving the combined action of the ERK and p38 pathways. Mol Immunol.

[88] Cai Z, Yang F, Yu L, Yu Z, Jiang L, Wang Q, Yang Y, Wang L, Cao X, Wang J (2012). Activated T cell exosomes promote tumor invasion via Fas signaling pathway. J Immunol.

[89] Ye SB, Li ZL, Luo DH, Huang BJ, Chen YS, Zhang XS, Cui J, Zeng YX, Li J (2014). Tumor-derived exosomes promote tumor progression and T-cell dysfunction through the regulation of enriched exosomal microRNAs in human nasopharyngeal carcinoma. Oncotarget.

[90] Chan JM, Ho SH, Tai IT (2010). Secreted protein acidic and rich in cysteine-induced cellular senescence in colorectal cancers in response to irinotecan is mediated by P53. Carcinogenesis.

[91] Weiswald LB, Guinebretiere JM, Richon S, Bellet D, Saubamea B, Dangles-Marie V (2010). *In situ* protein expression in tumour spheres: development of an immunostaining protocol for confocal microscopy. BMC Cancer.

